# Supplementary figures and images for: Comprehensive genomic characterisation of the NAC transcription factor family and its response to drought stress in Eucommia ulmoides
Source: PeerJ. 2023 Oct 23;11:e16298. doi: 10.7717/peerj.16298 (PMC10601904; doi:10.7717/peerj.16298)

## Supplementary Figures

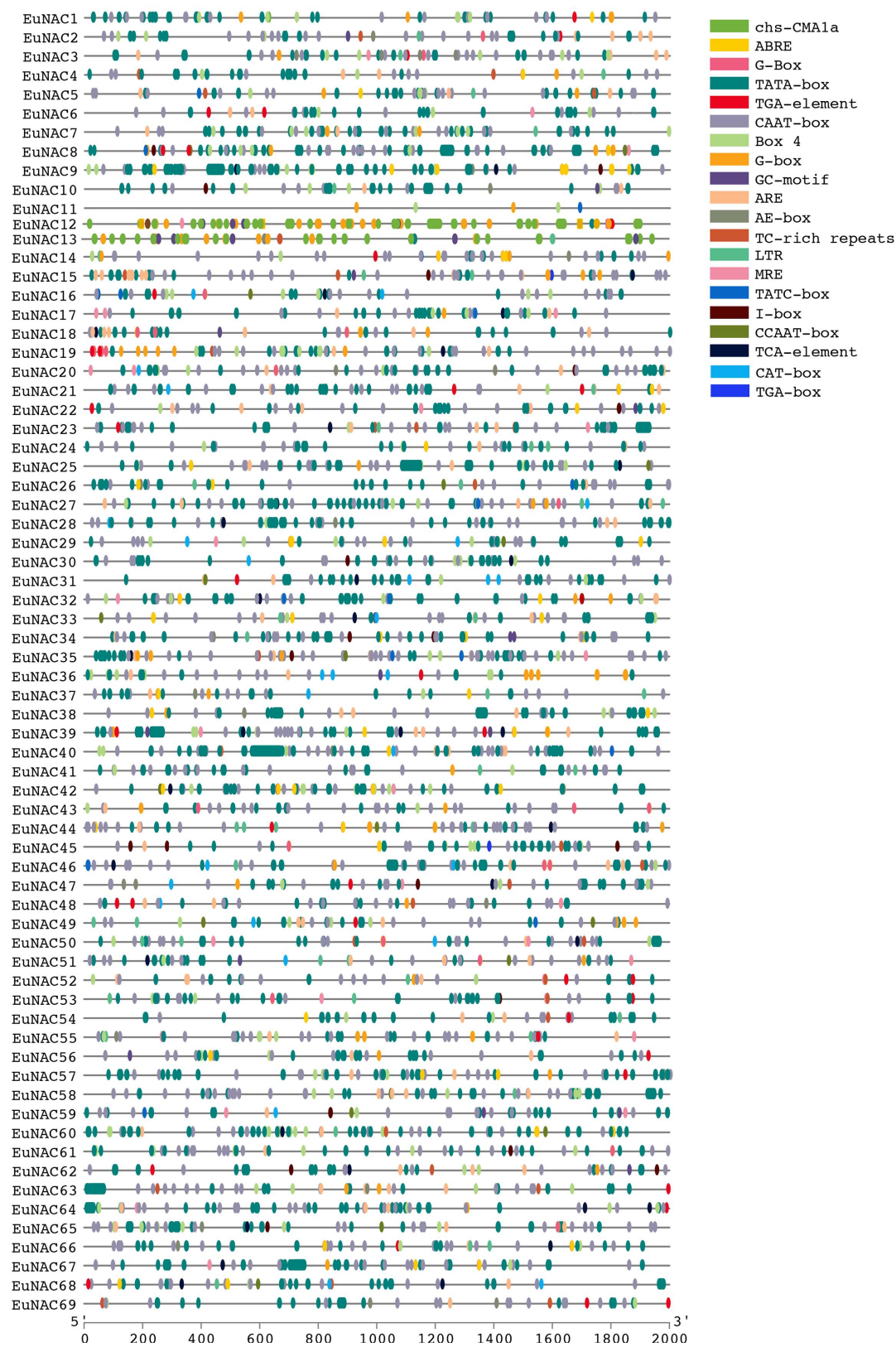

Figure S1. cis-acting elements in each EuNAC

Supplement: Supplemental Information 1 [file peerj-11-16298-s001.pdf]
